# Measuring Valve Gradients and Areas

**DOI:** 10.1016/j.jscai.2022.100433

**Published:** 2022-08-10

**Authors:** Larry S. Dean, Morton J. Kern

**Affiliations:** a University of Washington School of Medicine, Seattle, Washington; b VA Long Beach Health Care, Long Beach, California

**Keywords:** aortic valve area, Gorlin formula, Hakki formula, hemodynamics, mitral valve area

Although echocardiographic valve area determinations have taken the primary role in noninvasive assessment, it is important to remember that the standard on which echocardiography is based is the catheter-based hemodynamic data, which include aortic and left ventricular (LV) pressures and the measurement of cardiac output (CO). The original calculations of mitral and aortic valve stenosis have remained vital for >70 years.^1^

In brief, invasively obtained transvalvular pressure gradients and the calculation of valve areas require the following:

• An appropriate catheter and recording system for the accurate measurement of pressures^2^ above and below the valve under study
• Simultaneous transvalvular pressures should be obtained whenever possible. See alternatives to the dual lumen catheter^3^
• A hemodynamic recording system capable of calculating the mean gradient and valve area or one may use peak-to-peak gradients for aortic stenosis if a mean gradient is not available (see below)
• An accurate CO measurement
• An understanding of the underpinnings of the formulas that are used to make valve area calculations

## Valve area calculations from catheter-based pressures

There are 2 formulas that can be used to calculate valve area—the Gorlin and Hakki formulas. The gold (perhaps old as well) standard is the Gorlin formula, first published in 1951.^1^ Since then, other formulas have been proposed and, although a bit simpler, are all based on the original study by Gorlin and Gorlin.^1^

To understand the history, we must turn the clock back to the early beginnings of invasive cardiac catheterization and cardiovascular surgery. There was a need to assess valvular heart disease, predominantly rheumatic, at a time before cardiopulmonary bypass and echocardiography were available. One needed to know who would need surgery (done by closed mitral commissurotomy in the beating heart). The Gorlin formula was developed for this very reason.

Based on fundamental hydraulics, a valve area (cm^2^) was equal to flow (mL/s) across the valve divided by the square root of the pressure difference (ie, the gradient or ΔP) across the valve × 2 constants. The first constant is the discharge coefficient, an empirical constant of 1 for the aortic valve and 0.7 for the mitral valve (these were assigned arbitrarily to improve data fit). The second constant is 44.5, which is a blood acceleration factor (square root of 2 × gravity acceleration factor, 980 cm/s^2^). Only the flow across the valve during valve opening is used.

**Figure 1:**
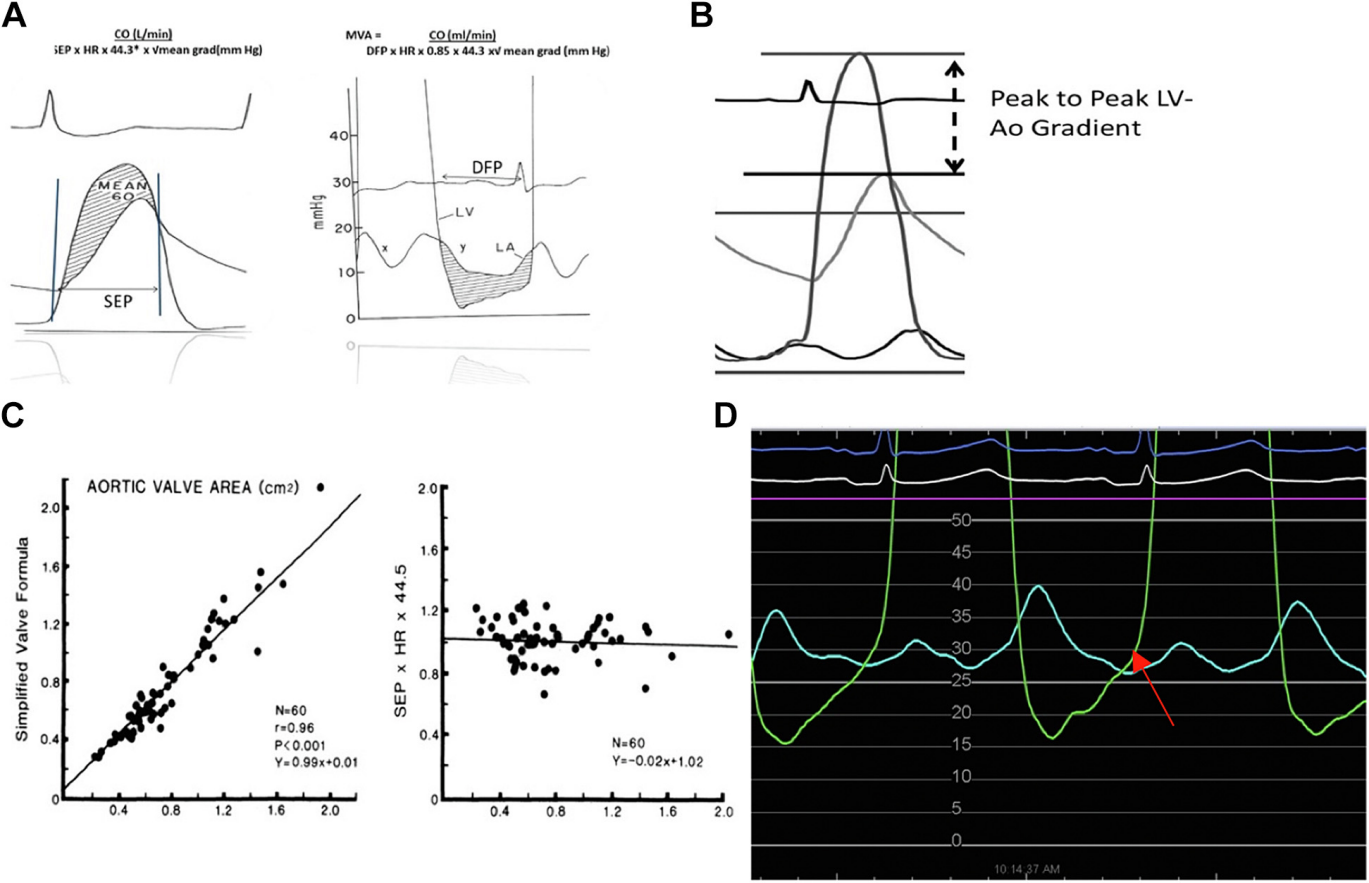
**Hemodynamics of aortic and mitral valve stenosis.** (**A**) Gorlin for AS. Aortic (left) and mitral (right) valve area calculations by the Gorlin formula. ∗44.5 = the square root of twice the gravity acceleration factor (980 cm/s^2^). The correct factor for mitral valve coefficient is 0.85. (**B**) Calculating AS by the Hakki formula using peak LV minus peak Ao pressures. (**C**) AVA by Gorlin formula and simplified (Hakki) formula. Left panel, correlation between Ao valve area by original Gorlin formula and simplified valve formula. Mean pressure difference was used in both formulas. Right panel, correlation between the product of SEP × HR × 44.5 and Ao valve area as measured by the original Gorlin formula. The SEP × HR × 44.5 is close to 1.^5^ (**D**) Simultaneous LV and pulmonary capillary wedge pressures in a patient with mechanical mitral valve replacement. Note the diastolic pressure gradient and importantly, the elevated left ventricular end diastolic pressure (arrow). Ao, aortic; AS, aortic stenosis; AVA, aortic valve area; CO, cardiac output; DFP, diastolic filling period; HR, heart rate; LA, left atrium; LV, left ventricle; MVA, mitral valve area; SEP, systolic ejection period per beat; SEP × HR, aortic valve flow during systole.

For aortic stenosis, flow was equal to CO (mL/min) divided by the fraction of flow for each heartbeat during systole (ie, heart rate [HR, beats/min] × the systolic ejection period [SEP, point of aortic valve opening on LV curve to the dichotic notch of aortic valve closure]) (Figure 1A, left side).

For mitral stenosis, flow was equal to CO (mL/min) divided by the fraction of flow during diastole (ie, HR × the diastolic filling period [point on the LV curve where the mitral valve opens to end of the “a” before isovolumetric contraction, s/beat]) (Figure 1A, right side). In 1972, Cohen and Gorlin^4^ revised the original formula and suggested the use of 0.85 for the mitral valve (instead of 0.7) as the discharge coefficient. The modern Gorlin formula is as follows:

Aortic valve area = CO (mL)/(SEP × HR × 44.3 × √mean gradient)
Mitral valve area = CO (mL)/(DFP × HR × 0.85 × 44.3 × √mean gradient), where DFP = diastolic filling period

## Key points for accurate calculations

• Accurate valve area calculation requires accurate measurement of CO. Note that in low flow states, valve areas may be underestimated.
• For low flow states, dobutamine can be used to increase the CO and hence the gradient for better assessment.
• Gorlin formulas use mean gradients and not peak gradients (which are used for the Hakki aortic stenosis formula, see below).
• The calculated valve area does not depend on the HR in the same way that echocardiographic calculations require because HR and the mean gradient are intrinsic to the Gorlin formulas.
• With irregular rhythms, such as atrial fibrillation, averaging the data over 10 beats rather than using a single beat is more accurate.
• Significant valvular regurgitation will cause an underestimation of the valve area because routine CO measurements do not account for the true flow across the valve (CO in valvular regurgitation is usually higher than actually measured).
• Unlike echocardiographic measurements, the left ventricular end diastolic pressure (LVEDP) is actually measured, which can potentially “unmask” diastolic dysfunction.

For example, consider a mean mitral valve gradient of 30 mm Hg with an LVEDP of 20 mm Hg. Following successful balloon valvuloplasty, the mean mitral valve gradient is reduced to 5 mm Hg but the LVEDP is still 20 mm Hg. No wonder your patient still has symptoms! (Figure 1D)

## The Hakki formula for valve areas

In 1981, A-Hamid Hakki, a cardiology fellow at Hahnemann University, after planimetering 60 aortic valve tracings and 40 mitral valve tracings, proposed that the Gorlin formula should be simplified because the factor HR × SEP × 44.3 (the Gorlin constant) was always “1.” Hakki et al^5^ then published the simplified Gorlin formula, using the peak LV–peak aortic pressures (or mean). The Hakki valve area formula is as follows:

AVA_Hakki_ or MVA_Hakki_ = CO/ √ peak − peak (LV-Ao) or mean (mitral) gradient, where AVA = aortic valve area, MVA = mitral valve area, and Ao = aortic (Figure 1B)

Hakki data have a very strong correlation to the Gorlin formula (Figure 1C).^6^ It remains highly accurate except in patients with significant bradycardia (<60 beats/min) or tachycardia (>100 beats/min).

**Pearls in brief**

Pearl #1: Invasive hemodynamics of valvular stenoses are the gold standard on which other techniques, such as echocardiography, are based.

Pearl #2: Accurate assessment of CO is critical. Underestimation of CO is a major pitfall in calculations when there is significant valvular regurgitation.

Pearl #3: An understanding of the formulas used to calculate valve area allows one to not only interpret the data but also to understand when there is an issue with the accuracy of the measurement and how to troubleshoot the problem.

Pearl #4: Do not forget the importance of knowing diastolic function (LVEDP) in the care of your patients post percutaneous or surgical valve procedures.

## Declaration of competing interest

The author(s) declared no potential conflicts of interest with respect to the research, authorship, and/or publication of this article.

## Funding sources

This research did not receive any specific grant from funding agencies in the public, commercial, or not-for-profit sectors.
